# Platelet-Activating Factor (PAF) in Allergic Rhinitis: Clinical and Therapeutic Implications

**DOI:** 10.3390/jcm8091338

**Published:** 2019-08-29

**Authors:** Rosa M. Muñoz-Cano, Rocio Casas-Saucedo, Antonio Valero Santiago, Irina Bobolea, Paula Ribó, Joaquim Mullol

**Affiliations:** 1 Allergy Section, Pheumology & Allergy Department, Hospital Clinic, Barcelona, 08036 Catalonia, Spain; 2 Clinical & Experimental Respiratory Immunoallergy, Institut d’Investigacions Biomediques August Pi I Sunyer (IDIBAPS), Barcelona, 08036 Catalonia, Spain; 3 ARADyAL, Instituto de Salud Carlos III, 28029 Madrid, Spain; 4 CIBER of Respiratory Diseases (CIBERES), Instituto de Salud Carlos III, 28029 Madrid, Spain; 5 Rhinology Unit & Smell Clinic, ENT Department, Hospital Clinic, Barcelona, 08036 Catalonia, Spain

**Keywords:** allergic rhinitis, anaphylaxis, asthma, epinastine, nasal congestion, platelet-activating factor, ketotifen, PAF antagonist, rupatadine

## Abstract

Platelet-activating factor (PAF) is a lipid mediator involved in several allergic reactions. It is released from multiple cells of the immune system, such as eosinophils, neutrophils, and mast cells, and also exerts its effect on most of them upon specific binding to its receptor, becoming a pleiotropic mediator. PAF is considered a potential relevant mediator in allergic rhinitis, with a key role in nasal congestion and rhinorrhoea due to its effect on vascular permeability. Interestingly, despite its potential relevance as a therapeutic target, no specific PAF inhibitors have been studied in humans. However, rupatadine, a second-generation antihistamine with dual antihistamine and anti-PAF effects has shown promising results by both blocking nasal symptoms and inhibiting mast cell activation induced by PAF, in comparison to antihistamine receptor drugs. In conclusion, the inhibition of PAF may be an interesting approach in the treatment of allergic rhinitis as part of a global strategy directed at blocking as many relevant inflammatory mediators as possible.

## 1. Introduction

**Figure 1:**
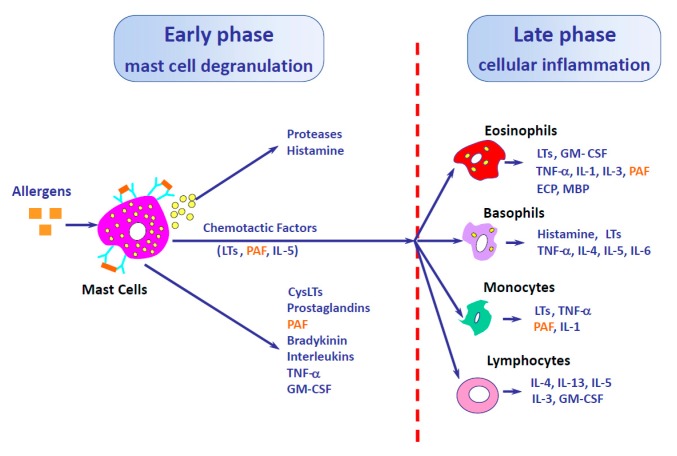
Importance of platelet-activating factor (PAF) in the early and late phases of allergic response. Adapted from [4] with permission.

Platelet-activating factor (PAF) was originally described in 1974 by Jacques Benveniste as a mediator released by basophils in an IgE-dependent manner, capable of inducing platelet aggregation [1]. PAF is a lipid mediator synthesized in two steps. First, membrane phosphocholine is transformed into lyso-PAF by the actions of cytosolic phospholipase A2; after that, PAF is synthesized from its precursor, lyso-PAF, by acetyl-CoA lyso-PAF acetyltransferase [2]. PAF can be rapidly released (30 s) upon cell stimulation, but it is also produced in the late phase of allergic reactions (Figure 1) [3,4]. PAF has a short half-life (3–13 min) and its degradation is catalyzed by PAF acetyl-hydrolase (PAF-AH) [5]. PAF plasma levels are maintained as low as 54 ± 40 pg/mL in healthy individuals in order to maintain the homeostatic functions [6], but are increased in some diseases such as liver cirrhosis, disseminated intravascular coagulation [6] or acute anaphylaxis [7,8]. PAF-AH deficiency has been related to allergic diseases, and a correlation between PAF-AH serum levels and anaphylaxis severity has also been described [8].

**Figure 2:**
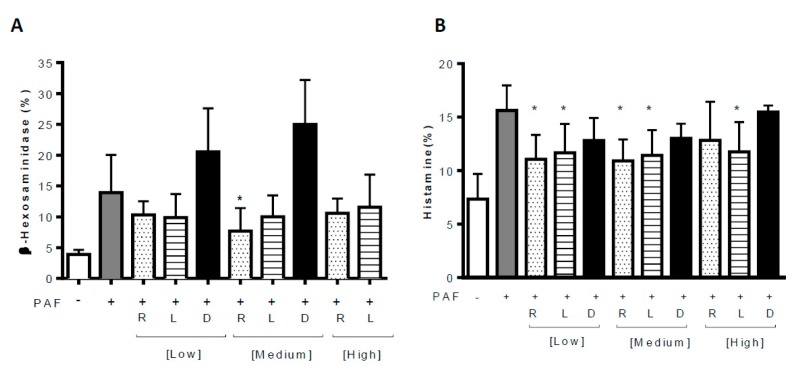
Effect of rupatadine, levocetirizine and desloratadine on PAF-induced (**A**) β-hexosaminidase and (**B**) histamine release in LAD2 cell line. R: rupatadine; L: levocetirizine; D: desloratadine. [Low]: 5 µM, [Medium]: 10 µM, [High]: 25 µM. * *p* < 0.05. (+) experimental condition with PAF. (−) experimental condition without PAF.

PAF is released by several cell types, including mast cells, eosinophils, platelets, neutrophils, monocytes, basophils, epithelial and endothelial cells [9]. Indeed, most of these cell types express PAF receptors (PAF-r) [10]. PAF-r is a seven-transmembrane G-protein-coupled receptor that, following activation, becomes rapidly desensitized. This refractory state is dependent on PAF-r phosphorylation, internalization, and down-regulation [9]. PAF-r binding induces different effects, and mediators release depending on the activated cell type or its characteristics. While PAF induces histamine and PGD_2_ release in human lung mast cells (MC) or MC derived from peripheral progenitors, it has no effect in human skin MC due to a lack of PAF-r expression [11]. In eosinophils, PAF is an extremely potent chemoattractant and promotes chemokine generation, as well as eosinophils activation or prostaglandin production (Figure 2) [12,13]. The ability of PAF to promote eosinophil migration is significantly increased in asthmatic subjects in comparison to healthy individuals [14]. Neutrophils are major producers of PAF [15], which is released in vitro upon FcγR binding (IgG receptor) [16]. Several studies have reported that neutrophils, in an IgG-dependent manner and with PAF as a main mediator, are involved in mouse anaphylaxis [17,18], but also in humans [19]. PAF is also a neutrophil chemoattractant, and some studies have shown neutrophil recruitment into the human nasal airway after a nasal challenge with PAF, although this recruitment is faster in atopic individuals, thereby suggesting a different sensitivity to PAF effects [20] (Figure 1).

## 2. Role of PAF in Allergic Diseases

### 2.1. PAF in Allergic Rhinitis

The role of PAF in allergic rhinitis (AR) has also been suggested. PAF is considered the strongest inducer of vascular permeability, and therefore plays a key role in rhinorrhoea and nasal congestion [21,22]. Similar to asthma, increased levels of both PAF and its precursor lyso-PAF have been found in nasal lavages and plasma samples in AR patients [23]. Indeed, nasal challenge with allergens (pollen) has been shown to increase lyso-PAF and PAF-AH levels in nasal lavage samples, peaking at 10 min and returning to baseline levels at 60 min [23]. Nasal challenge with PAF, similar to asthma, reproduces rhinitis symptoms, and also decreases nasal patency, increases eosinophilic and neutrophilic infiltration, as well as nasal hyperreactivity [20,22,24]. It has also been proposed that PAF plays a role in the priming phenomenon, understood as the influence of one stimulus to a subsequent stimulus (enhancing its effect). In line with that, there are studies showing a greater nasal response after nasal challenge with histamine or bradykinin, if PAF has been administered previously [25]. PAF receptors have recently been found expressed in lung human mast cells [26] as well as in healthy and inflamed upper airway mucosa [27].

Contradictory conclusions regarding the differential effects of PAF in AR and healthy individuals can be found in the literature. Whereas some authors, such as Klementsson et al. [28] have only observed symptoms in AR patients after nasal challenge with PAF, others such as Leggiere et al. [25] and Muñoz-Cano et al. [22] have demonstrated an effect in both AR and healthy individuals. This discrepancy could highlight an interesting aspect, because, as seen in other diseases and models, allergic patients may be more sensitive to the effect of PAF than healthy individuals [29]. Muñoz-Cano et al. [22] observed that the symptoms in allergic patients, measured using a Likert and visual-analogue scale (VAS), were more intense than in the healthy control group, although the differences were not statistically significant. However, none of the published studies directly address the possible differences in the sensitivity to PAF.

There are several studies using PAF nasal challenges aiming to unravel the pathogenesis of AR. Nasal challenge with PAF induces AR symptoms, and its peak is reached 30–120 min after PAF instillation and lasts up to 240 min [22,25,28]. The symptoms’ peak depends on the dose and schedule of the PAF used for the challenge. Most studies use a single dose of PAF, ranging from 30 to 600 nM, observing the peak at 30 min [25,28]. Another study, using progressively increasing doses (100 nM, 200 nM, 400 nM every 30 min), with a cumulative dose of 700 nM, observed the symptoms peak at 60 min after the last dose (120 min after the first dose) [22]. These discrepancies are difficult to explain. Considering PAF’s priming effect, the study using the cumulative schedule should have observed the symptoms in an earlier time point, compared to the single dose schedule. However, the magnitude of the symptoms may be different depending on the dose. Therefore, the “peak” observed in one study may be lower than the “peak” of another study that uses higher doses of PAF. For the same reason, depending on the concentration of PAF used for the nasal challenge, the duration of the effects may be different. However, Leggieri et al. [25] observed almost a resolution of the symptoms, just 60 min after instillation, of 600 nM of PAF. Muñoz-Cano et al. [22], conversely, with a similar dose (700 nM), observed it 240 min after instillation. Although those two studies had similar doses, they each used a different schedule, namely single dose vs. cumulative. Therefore, in the single dose study the effect of PAF vanished rapidly after its instillation, whereas in the cumulative schedule the effect last 240 min after the first dose and 90 min after the last one, suggesting a priming effect.

PAF has been demonstrated to induce a wide range of nasal symptoms, but nasal congestion seems to be one of the most important. Actually, in one study the authors only observed nasal blockage, but no sneezing or itching, and a very mild rhinorrhoea [22]. That means that nasal congestion seems to be strongly related to PAF, although the role of other mediators needs to be considered. This is a good therapeutic opportunity, considering that nasal blockage is the most bothersome symptom in AR and antihistamines has a limited effect, and nasal corticosteroids are often then required to treat this symptom [30]. Although nasal corticosteroids are safe and very useful drugs in AR [31], there is a lack of adherence to the treatment, mostly related to corticophofia and some local side effects [32]. Therefore, the development of drugs targeting PAF may be an alternative to nasal corticosteroids for the treatment of the nasal congestion in AR patients.

Despite the observed involvement of PAF in AR, the PAF receptor antagonist has only been studied in animal models (with promising results), and no human studies have been performed [33,34]. However, there are several studies with rupatadine, a second-generation antihistamine, that has shown a dual effect on H_1_ histamine and PAF receptors [35,36,37]. Rupatadine has been successfully used in patients with allergic rhinitis (and urticaria), and clinical trials have demonstrated its safety and efficacy [4,37,38]. A systematic review/meta-analysis examined the efficacy of rupatadine by pooling data from ten randomized, double-blind, placebo-controlled studies involving more than 2500 patients. This meta-analysis showed that rupatadine was significantly better than the placebo in controlling allergy rhinitis, mainly nasal congestion as well as conjunctival symptoms. As a result of this analysis, a robust level of evidence was found, and a recommendation for use of rupatadine in allergic rhinoconjunctivitis was made.

**Figure 3:**
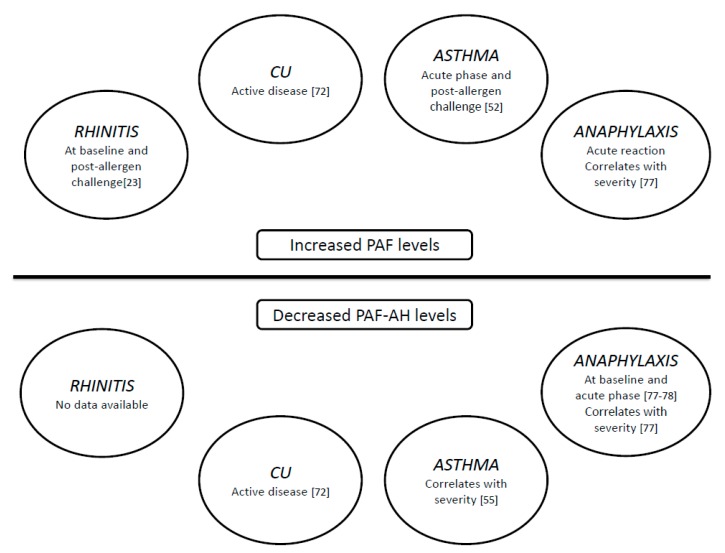
PAF and PAF-AH levels in respiratory diseases, anaphylaxis and chronic urticaria.

Rupatadine has demonstrated several anti-allergic effects, blocking mast cell degranulation, eosinophil and neutrophil chemotaxis, as well as cytokine production (IL-5, IL-8, GM-CSF and TNF-α) [4,38]. In order to demonstrate that the combined inhibition of PAF and histamine may be a better strategy to treat patients with AR, Muñoz-Cano et al. [39] designed a proof of concept randomized, double-blind crossover placebo-controlled study. Nasal challenge with PAF was performed in patients that were previously treated (during 4 days) with levocetirizine (H_1_ antagonist) or rupatadine (H_1_ and PAF antagonist). They compared seasonal allergic rhinitis (grass or tree pollen allergy) patients with healthy individuals, out of pollen season. The results showed that rupatadine, but not levocetirizine, significantly reduced PAF-induced symptoms in patients with AR (Figure 3). Rupatadine produced a significant 54% reduction in the area under the curve for the total 4 nasal symptoms scores (T4SS) induced by PAF, which includes mainly nasal congestion and rhinorrhoea, and in smaller proportion, itching and sneezing. Rupatadine caused a 73% decrease of the T4SS at 60 min after nasal challenge, compared to the 23% decrease observed with levocetirizine.

The same authors, using an in vitro approach, showed again that rupatadine, but not any of the other tested antihistamines (without PAF effect), significantly inhibited PAF-induced mast cell activation [26]. Mast cells from the human cell line LAD2 and primary human lung mast cells were incubated with PAF and either rupatadine, levocetirizine, desloratadine, PAF receptor antagonists CV6209, BN52021 or WEB2086. Mast cell degranulation was evaluated using both β-hexosaminidase and histamine assays. Rupatadine inhibited LAD2 β-hexosaminidase release, but neither levocetirizine nor desloratadine showed any significant effect (Figure 2A). Interestingly, levocetirizine inhibited histamine release in LAD2, similar to rupatadine (Figure 2B). Finally, in lung mast cells, only rupatadine blocked mast cell degranulation. Among the PAF receptor antagonists, BN52021 and WEB2086 failed to show any inhibitory effect, whereas CV6209 inhibited mast cell degranulation (both histamine and β-hexosaminidase) in all the tested cell types. Finally, these authors suggest a comparable effect on inhibiting mast cell activation when comparing rupatadine and CV6209.

Kajiwara et al. [11] showed a similar activation pattern, but used a lower concentration of PAF (1nM vs. 10 µM in Muñoz-Cano et al.). The different cell types used (progenitor-derived mast cells vs. LAD2 or human lung mast cells) in the studies may account for those differences. Similar to the previous study, the compound CV6209 also demonstrated an inhibitory effect on PAF-induced histamine release.

Finally, Alvezios et al. [40] demonstrated the inhibitory effect of rupatadine on PAF-induced histamine and cytokine release using the LAD2 model. Those authors compared rupatadine with an old antihistamine with no PAF effect (diphenhydramine). Indeed, rupatadine, but not diphenhydramine, inhibited mast cell mediator release.

Several other antihistamines have also shown, in vitro, an anti-PAF effect including azelastine, oxatomide, ketotifen and epinastine. Shindo et al. showed that both azelastine [41] and oxatomide [42] inhibited PAF release in neutrophils obtained from either asthmatic patients and healthy individuals. Other authors have demonstrated that epinastine [43] and ketotifen [44] suppressed rabbit platelet aggregation induced by PAF at higher concentrations. Ketotifen also inhibited beta-hexosaminidase release from mouse mast cells derived from bone marrow [45].

In summary, PAF plays an important role in allergic rhinitis, and in contrast to the observation in asthma, blocking this mediator seems to improve rhinitis symptoms, and therefore may be considered a therapeutic target. However, PAF is only one of the several inflammatory mediators involved in allergic rhinitis. In our opinion, we propose that anti-PAF drugs may contribute to a better control of nasal symptoms, such as nasal congestion or rhinorrhea, compared to the use of antihistamines with no other anti-inflammatory properties. The in vivo studies using the only commercially available antihistamines with known anti-PAF activity, such as rupatadine [38], azelastine [46], ketotifen [47] or epinastine [48], have demonstrated their efficacy and safety as a daily treatment for allergic rhinitis. Among those, rupatadine is the only compound that has demonstrated their capacity for an inhibition of PAF release in nasal airways, in several in vitro and in vivo studies. This may suggest that PAF blockage may be usefully included in a strategy aimed at inhibiting as many mediators as possible. However, further clinical studies are needed with anti-PAF compounds in AR patients.

### 2.2. PAF in other Respiratory, Cutaneous and Allergic Diseases

#### 2.2.1. PAF in Asthma

Current evidence suggests that PAF plays a role in the immune and inflammatory response in asthma. Several studies have shown that PAF, in bronchial challenge, induces bronchoconstriction and increases airway hyperreactivity [49]. Moreover, it has also been shown that PAF favors mucus production and increases vascular permeability of pulmonary blood vessels [50]. Interestingly, inhaled PAF stimulates the production of cysteinyl leukotrienes, and it is likely that the effects of PAF in the lungs may be mediated by some of these products [51]. PAF is produced in response to several pro-inflammatory stimulus, such as allergens but also during infections [12]. Increased PAF levels in sputum and bronchoalveolar lavage samples of asthmatics patients have been found during an asthma attack and after allergen challenge [52]. Interestingly, smooth muscle proliferation induced by salbutamol is mediated by PAF. Indeed, salbutamol induces PAF synthesis and inhibits its catabolism [53]. It has also been speculated that PAF may inhibit the response to beta-adrenergic agonists [54].

Furthermore, similar to anaphylaxis, PAF-AH levels inversely correlate with asthma severity in Japanese children; a missense mutation in the PAF-AH gene results in inactive PAF-AH and occurs in 4% of the Japanese population [51]. Stafforinie et al. found that a deficiency in PAF-AH may constitute a risk factor for the development of asthma and also asthma attacks, being the subjects with a complete deficiency, at risk of developing severe asthma [55].

Even though it is the suggested role of PAF in this disease, most PAF receptor inhibitors have failed to show any significant beneficial effect in asthma symptoms in humans [51]. Y-24180, a PAF receptor antagonist, improved bronchial hyperreactivity measured by methacholine. However, the randomized clinical trials failed to demonstrate its utility as a routine asthma treatment [51]. Another PAF antagonist, WEB2086, could not attenuate either the early or the late asthmatic response induced by an allergen. No effects on lung function, rescue medication or inhaled corticosteroids dose were observed [56]. Regrettably, several studies with different PAF antagonists had the same outcomes as the previously described, not showing sufficient beneficial effects for the regular treatment of asthmatic patients [51].

Considering that PAF-AH deficiency may be related to asthma and its severity, another interesting approach could be the administration of recombinant PAF-AH. Although promising results in the murine models and the pre-clinical studies showed a decrease of inflammation, PAF-AH failed to attenuate neither the early nor the late allergic response in mild asthma patients [57,58].

There is not only one inflammatory mediator involved in asthma, and therefore, strategies targeting PAF and other mediators as histamine have also been considered. Azelastine, oxatomide and epinastine, dual PAF and histamine antagonists, have shown a remarkable effect of blocking PAF-induced bronchoconstriction in guinea pigs and rats [59,60,61] and its use is approved for asthma patients in Japan as an add-on treatment [62]. Ketotifen, also included in the Japanese asthma guidelines [62], has shown to improve asthma control and wheezing in children with mild and moderate asthma [63]. Conversely, the studies with another dual antagonist, Sch37370, showed an effective blockage of the bronchospasm induced by histamine, PAF, antigen or serotonin, as well as antigen-induced eosinophilia, in guinea pigs [64]. However, as far as we know, no studies in humans have been performed. Finally, rupatadine has been successfully used in rhinitis and urticaria patients, but not in asthmatics [4,37].

In conclusion, despite the important role of PAF in asthma, the blockage of this mediator alone does not seem to have a significant effect on asthma symptoms. Considering the complex networks and signaling pathways involved in asthma pathogenesis, PAF seems to be just a downstream mediator. It may be, for this reason, that corticosteroids are still the first-line treatment, because of its capacity of blocking upstream signals, and therefore, creates the effect of multiple mediators on multiple cells types and tissues [65].

#### 2.2.2. PAF in Chronic Urticaria and Food Allergy

Chronic urticaria (CU) is an inflammatory skin disorder, which is defined by recurrent itchy wheals, associated or not with angioedema, and would have been continuously or intermittently present for at least 6 weeks [66]. In most cases, chronic urticaria is related to idiopathic mechanisms, and in some cases, to immunological non-allergic and exceptionally to allergic mechanisms [66,67]. Mast cells are considered the most important cells in the pathogenesis of CU, and histamine is the predominant mediator [68]. However, other cell types such as basophils [69], and mediators such as cysteinyl leukotrienes, serotonin, TNF-α and PAF are also thought to play a part in urticaria [12,66,68]. PAF effects on urticaria seem to be related to the increase of vascular permeability, particularly in skin capillaries, enhancing the effect of other mediators and the development of wheals [12,21,70]. A recent study published in 2019 by Ullambayar et al. [71] has shown that patients with spontaneous CU have higher levels of PAF and lower levels of PAF-AH, the enzyme degrading PAF, in comparison to a group of healthy individuals. Those authors also compared a subgroup of CU patients who had not responded to antihistamines (up to fourfold) after 3 months, with a group of patients who were responding to the antihistamine. Interestingly, they found that non-responders had higher PAF levels and lower PAF-AH levels. Finally, a multivariate analysis showed that PAF serum levels >5000 pg/mL could be a significant predictor of a poor response to antihistamine treatment.

Another interesting result from the Ullambayar study was the inverse correlation found between PAF-AH and the urticaria duration [71]. This suggests that PAF-AH may be a part of a compensatory mechanism that could explain why there is a resolution of the symptoms in up to 50% of CU patients after 3 years of symptoms [72]. At some point, PAF-AH may increase enough to block the effect of PAF, reducing the PAF-related enhancement of histamine release and therefore resolving the urticaria symptoms. However, there is a need for studies to assess PAF-AH levels at different time points in CU patients, comparing those who achieve a symptom-free situation with those that are still symptomatic after 3 years.

Finally, a further interesting analysis in the Ullambayar study showed a positive correlation between triglyceride, total cholesterol and body mass index (BMI), and PAF-AH levels, only in the CU patients [71]. The active form of PAF-AH, also known lipoprotein-associated phospholipase A_2_, circulates as a complex with low-density lipoproteins (LDL) [5]. Plasma PAF-AH concentration has been shown to directly correlate with LDL levels in a cohort of 240 normolipidemic individuals [73]. This means that lowering LDL in plasma results in reduced levels of PAF-AH, and therefore, a longer half-life for PAF [73], that could increase the risk for severe anaphylaxis or, perhaps, other allergic reactions. This relationship between cholesterol metabolism and PAF has previously been described by Perelman [7] in a group of children who demonstrated peanut allergy. They studied 63 children between 2–19 years that had urticaria with or without angioedema related to peanuts, with positive skin tests (≥8 mm wheal) and/or peanut-specific IgE ≥ 14 kU/L. A strong correlation between PAF-AH and plasma concentration of Apoprotein B, considered as a good surrogate to measure LDL concentration [5], was found. However, no healthy individuals were used as a control, thereby limiting the interpretation of these results.

#### 2.2.3. PAF in Anaphylaxis

Anaphylaxis is a severe, immediate, life-threatening reaction often related to allergic mechanisms, i.e., food, drug or insect allergy. There are no treatments to prevent these reactions, and only drugs for acute management are available [72]. Several mediators are released during an anaphylactic reaction from several cell types, although mast cells and basophils are the principal cells involved in those reactions [74]. Tryptase is protease mostly released by mast cells, widely used as a marker of mast cell activation and clonal expansion [75]. However, neither tryptase nor histamine correlates with the severity of anaphylactic reactions. Interestingly, Vadas et al. showed that PAF, compared to tryptase and histamine, correlates more accurately with anaphylaxis severity [76]. PAF is related to the increase of the vascular permeability; therefore, these authors compared PAF levels in patients who developed angioedema during anaphylaxis, with those that had hypotension, and found higher levels in the latter patients.

In 2008 Vadas et al. showed, for the first time, the positive correlation between anaphylaxis severity and PAF levels, and the negative correlation between severity and PAF-AH levels. The authors compared PAF and PAF-AH levels in patients with fatal peanut anaphylaxis, healthy controls (both adult and children), children with peanut allergy (urticaria/angioedema) at baseline, and non-fatal peanut acute anaphylaxis. Fatal anaphylaxis was related to the lowest PAF-AH levels, but there were no differences between the other groups. However, the number of patients with a decrease PAF-AH activity (≤20 nmol/mL/min) was higher in the anaphylaxis group, and most importantly, in grade 3 anaphylaxis individuals. Surprisingly, there is no data regarding PAF or PAF-AH levels at baseline in the anaphylaxis group. It could be that the patients with more severe anaphylaxis may have lower baseline levels of PAF-AH and this could thus be used as a biomarker. A study from Pravettoni et al. [77] addressed this matter in a group of patients allergic to hymenoptera venom. They found that basal PAF-AH levels inversely correlated with anaphylaxis grades, suggesting that PAF-AH levels may predict anaphylaxis severity in the future.

The relevance of PAF in anaphylaxis was demonstrated in a PAF receptor knockout (KO) mouse model [78]. PAFr KO mice suffered from less severe anaphylaxis, none of them fatal, compared to normal animals. The authors suggested that, among the different mediators involved in an anaphylaxis that includes histamine or eicosanoids, PAF has the most important role. Similarly, Kajiwara et al. [11] suggested that the relevance of PAF in anaphylaxis may be related to its capacity to amplify the allergic reaction (from local to systemic). Also using a mouse model, Jordana et al. showed that PAF antagonists significantly attenuated the magnitude and duration of the anaphylaxis [17]. However, neither histamine nor leukotriene blockage significantly affected any of those parameters. While 83% of PAF antagonist-treated mice recovered within 120 min after the peanut challenge, only 43% of the untreated ones did it, and 50% of the treated mice developed either no or mild anaphylactic reactions. Indeed, a greater protective effect was achieved when using a dual strategy, blocking histamine and PAF, compared to blocking PAF alone. The beneficial effect of a concomitant blockade of multiple mediators has previously been demonstrated by Brandt et al. [79], blocking PAF and serotonin in an ovalbumin-induced allergic diarrhea model. Actually, it was also observed in the Jordana et al. [17] model that combined therapy was associated with a significant decrease in vascular leakage and release of vasoactive mediators.

## 3. Conclusions

Compared to the research performed in asthma, PAF has been less studied in AR and its potential role as a therapeutic target is therefore less known. This lack of strong evidence, and the failure of PAF inhibition as a therapeutic strategy in asthma, may account for the lack of interest in performing studies with PAF inhibitors in AR. However, the failure of PAF inhibition in asthma does not necessarily correlate with a lack of efficacy in AR. Antihistamines are widely and effectively used in AR and are useless in asthma. So, despite the concept of “one airway, one disease” still being valid, some drugs may target the upper and the lower airways differently, and therefore, be useful in some diseases and not in others. Further research is needed in the field of AR, because it seems obvious that targeting more than one inflammatory mediator would provide a better control of the inflammation, and therefore, of the symptoms.

A summary of PAF actions, and PAF and PAF-AH profile in different diseases has been pictured in Table 1 and Figure 3, respectively.

## Figures and Tables

**Table 1:** PAF in allergic diseases.

Allergic Rhinitis	
In vivo effects	Reproduces rhinitis symptoms (PAF concentration 10 to 500 nmol) [20,22,25,39] Increases vascular permeability (associated with rhinorrhea and nasal congestion) [21] Nasal hyperreactivity [20,22,24] Priming phenomenon [25]
In vitro findings	PAF receptor is expressed in multiple cell types (mast cells, eosinophils, platelets, endothelial cells, basophils, neutrophils, epithelial cells, etc.) [9] Potent chemoattractant (eosinophils and neutrophils) [12,13]
Treatments	No PAF antagonists available for AR [33,34] Dual histamine and PAF antagonists available: rupatadine, ketotifen, epinastine, azelastine, oxatomide, etc. [35,36,37,39,41,42,43,44,45]
**Asthma**	
In vivo effects	Induces bronchoconstriction, airway hyperreactivity and mucus production [49,50] Increases vascular permeability [50]
In vitro findings	Induces cysteinyl leukotrienes production [51] Mediates smooth muscle proliferation induced by salbutamol [52] Alteration of PAF and PAF-AH levels (see Figure 2)
Treatments	PAF antagonists have failed to show any beneficial effect on asthma [51,52] Some antihistamine with anti-PAF effect are approved for asthma in some Asian countries as an add-on treatment [62]
**Chronic Urticaria**	
In vivo effects	No available data of the direct effect of PAF in human skin PAF activates LAD2, lung mast cells and mast cells derived from peripheral progenitors [11,26] PAF does not activate skin mast cells [11]
In vitro findings	Increase vascular permeability enhancing the effect of other mediators and the development of wheals [12,70] Alteration of PAF and PAF-AH levels (see Figure 2)
Treatments	No PAF antagonists are available Some dual antihistamines, such as as rupatadine [4], have shown greater control of symptoms and improvement of quality of life, compared to antihistamines with no anti-PAF effect as desloratadine
**Anaphylaxis**	
In vivo effects	No available data in humans PAF is the most important mediator compared to histamine or leukotrienes in several mouse models [17,78] Induces vascular leakage and release of vasoactive mediators [17,78]
In vitro findings	Alteration of PAF and PAF-AH levels (see Figure 2)
Treatments	No PAF antagonists are available Strategies blocking PAF along other inflammatory mediators reduces severity of anaphylaxis in mouse models [17,79]
